# Moderated Mediation Model of Interrelations between Workplace Romance, Wellbeing, and Employee Performance

**DOI:** 10.3389/fpsyg.2017.02158

**Published:** 2017-12-14

**Authors:** Muhammad Aamir Shafique Khan, Du Jianguo, Muhammad Usman, Malik I. Ahmad

**Affiliations:** ^1^School of Management, Jiangsu University, Zhenjiang, China; ^2^The Department of Management Sciences, COMSATS Institute of Information Technology, Lahore, Pakistan; ^3^Warwick Business School, Coventry, United Kingdom

**Keywords:** workplace romance, psychological wellbeing, employee performance, gender, healthcare sector

## Abstract

In this study, first we examined the effect of workplace romance on employee job performance, and the mediatory role of psychological wellbeing in the relationship between workplace romance and employee performance. Then we tested the moderating effects of gender and workplace romance type – lateral or hierarchical – on the indirect effect of workplace romance on employee performance. Based on a survey of 311 doctors from five government teaching hospitals in Pakistan, we used structural equation modeling and bootstrapping to test these relationships. This study reveals that psychological wellbeing significantly fully mediates the positive relationship between workplace romance and job performance. Moreover, multi-group analysis shows that gender moderates the indirect effect of workplace romance on employee performance, where the indirect effect of workplace romance on employee performance is stronger for male participants. This study carries important implications, particularly for the policy makers and managers of healthcare sector organizations.

## Introduction

Workplace romance is understood as a mutually-welcomed (Pierce and Aguinis, 2003) and committed (Diamond et al., 1999) relationship between two members of an organization that may involve physical attraction and activities such as dating, hugging, kissing, touching, and sexual intercourse (Pierce et al., 1996; Mainiero and Jones, 2013). Workplace romance is argued to be an undeniable reality in the social, relational, and political fabric of a workplace (Quinn, 1977; Pierce et al., 1996; Wilson, 2015). Workplace romance affects a number of important employees’ work-related behaviors and attitudes such as work motivation, job satisfaction, commitment, work engagement and loyalty and performance outcomes (Quinn, 1977; Pierce and Aguinis, 2009). However, despite its essential presence in the workplace and important effects on employee behavior and performance, Kolesnikova and Analoui (2012) and Wilson (2015) rightly note that workplace romance remains the most ignored aspect of organizational life.

Additionally, workplace romance discourse is characterized by a lack of consensus on the nature of its effects on employee work-related behaviors and performance outcomes. Literature on this topic suggests that workplace romance can have both destructive and constructive effects on employee behavior and performance (Pierce and Aguinis, 2003; Wright and Cropanzano, 2007; Wilson, 2015; Baker, 2016). The common destructive outcomes of workplace romance include hostility, cynicism and disapproval from peers and managers, deterioration in employee productivity and performance, litigations and ethical issues (Williams et al., 1999; Burke, 2010). Conversely, the literature also suggests that workplace romance positively influence wellbeing, commitment, and employee performance (Dillard and Broetzmann, 1989; Pierce and Aguinis, 2003; Wright and Cropanzano, 2007; Biggs et al., 2012). The lack of consensus suggests that the relationship between workplace romance and employee performance has yet to be firmly established. Moreover, empirical studies on the relationship between workplace romance and performance are scarce.

The mixed theoretical perspectives also laud the workplace romance and employee performance relationship as being intricate in nature. The relationship can be mediated through, and affected by, a number of different behavioral and demographic aspects displayed by employees. For instance, workplace romance literature is indicative of the links between workplace romance and psychological wellbeing (Pierce et al., 1996; Hochschild, 1997; Cole, 2009; Biggs et al., 2012). We understand psychological wellbeing as “self-perceived success in important areas such as relationships, self-esteem, purpose, and optimism” (Diener et al., 2010, p. 143). Moreover, literature proposes a relationship between psychological wellbeing and employee performance (Iaffaldano and Muchinsky, 1985; Wright et al., 1993, 2007; Wright and Bonett, 1997; Judge et al., 2001; Moradi et al., 2014). This suggests that psychological wellbeing can mediate the relationship between workplace romance and employee performance.

Thus, one of the aims of this study is to first examine the effect of workplace romance on employee performance, and then test the mediatory role of psychological wellbeing in this relationship.

Furthermore, the aforementioned debate on the nature of the effect of workplace romance is linked to a number of different factors including gender and the type of workplace romance; i.e., lateral or hierarchical (Gutek, 1985; Powell and Foley, 1998; Biggs et al., 2012; Davis, 2012). This study expects that gender and the type of workplace romance moderate the interrelations between workplace romance, employee wellbeing, and employee performance.

Based on a survey of 311 doctors from five Pakistani public sector hospitals operating in the Punjab province of Pakistan, we used structural equation modeling (SEM), multi-group analysis, and bootstrapping to test these relationships.

This study is important in terms of theoretical contributions and practical implications. Studying workplace romance is imperative because of its effects on employee behavior and performance and the lack of consensus on the nature of these effects (Biggs et al., 2012). Our interest in psychological wellbeing is inspired by its important role in improving employee job involvement, performance and productivity (Brown and Leigh, 1996; Wright and Bonett, 1997; Judge et al., 2001; Wright et al., 2007). By studying the role of moderators – gender and romance type – in the relationship between workplace romance and employee performance, this study aims to contribute to the debate on the nature of the effects of workplace romance on employee behavior and performance.

Importantly, we contextualized the relationship between workplace romance, wellbeing and performance in healthcare settings. The job of healthcare professionals and particularly that of doctors plays a key role in determining the quality of human life. Thus, understanding the factors that affect the performance of doctors carries important practical implications. Moreover, studies on the relationship between workplace romance and performance in Muslim countries, including Pakistan, are rare.

## Hypotheses Development

### Workplace Romance and Performance

Traditionally, blending work-life and personal boundaries and becoming involved in romance in the workplace are portrayed as risky matters for employees as well as organizations (Mainiero and Jones, 2013; Baker, 2016). Workplace romance is argued as a way to foster conflicts between the right to privacy and professional codes of conduct (Mano and Gabriel, 2006; Mainiero and Jones, 2013). However, workplace romance scholarship is extending in scope. Quinn (1977) is largely credited with broadening the scope of workplace romance literature, as he brought to the fore the constructive as well as destructive dimensions of workplace romances. Quinn (1977) suggests that workplace romance can contribute to both the improvement and deterioration of different behavioral and performance-related outcomes of employees and organizations. That is, workplace romance can have both positive and negative effects on the behavioral and performance aspects of employees.

The Quinn’s conceptualization of workplace romance has drawn in researchers from different disciplines to debate and empirically study the relationship between workplace romance and the different facets of work-related behaviors and attitudes such as burnout, job satisfaction, commitment, absenteeism, wellbeing, and turnover intentions (Anderson and Fisher, 1991; Powell and Foley, 1998; Mano and Gabriel, 2006; Cole, 2009; Biggs et al., 2012; Davis, 2012; Wilson, 2015).

Literature has usually focussed on, and brought to the fore, several negative repercussions of workplace romance such as negative publicity, litigation and job withdrawals, hostility and cynicism (Williams et al., 1999; Burke, 2010; Wilson, 2015). The literature suggests that workplace romance negatively affects employee behavior and performance (Williams et al., 1999). Discussing the effects of workplace romance on nurses’ work and life, Sharma and Dhar (2016) posit that nurses’ exposure to patients with different types of mental and physical health issues, patients’ relatives, severe illness and death is an everyday occurrence. In such situations, workplace romance can result in additional stress for the romance participants and can distract them from work (Cole, 2009). For instance, workplace romance participants can face disapproval and criticism from peers, particularly if workplace romances are perceived to be detrimental for justice and equity (Cole, 2009). Workplace romance participants can draw different managerial reactions such as transfer, written warning and verbal reprimand if romances violate the prescribed patterns of behavior, practice, and performance (Dillard et al., 1994; Pierce and Aguinis, 2009). Such reactions from peers and managers can expose workplace romance participants to stress and lower their morale (Dillard et al., 1994; Cole, 2009; Pierce and Aguinis, 2009).

On the contrary, we expect a positive relationship between workplace romance and employee performance. This expectation is built on the following arguments. First, the impression management hypothesis proposes that workplace participants work hard and demonstrate improved performance to create a favorable impression (Dillard and Broetzmann, 1989; Pierce and Aguinis, 2003). The literature suggests that employees involved in workplace romances exhibit improved job satisfaction and performance (Wright and Cropanzano, 2007; Biggs et al., 2012). Gutek (1985) suggests that employees may take pleasure in gaining colleagues’ sexual attention. Consequently, workplace romance participants feel desirable and accepted in the workplace (Gutek, 1985; Pierce, 1998; Biggs et al., 2012).

Second, workplace romance literature has usually focussed on sexual or romantic behavior and sexual relations while studying the workplace romance relationship with employee behavior and performance (e.g., Pierce, 1998; Pierce and Aguinis, 2003; Mano and Gabriel, 2006; Burke, 2010; Salvaggio et al., 2011). Particularly, the operationalization of workplace romance construct in contemporary literature confines the scope of workplace romance and its behavioral and performance outcomes. For instance, Pierce and Aguinis (2003) measured workplace romance using one statement “I am currently romantically involved with (e.g., dating, married to) a member of my organization (1 = Yes, 0 = No).” Salvaggio et al. (2011) used self-reports and third-party reports to measure workplace romance. For self-reports, they adopted above mentioned single item. For the third-party reports, they used one item “Have you ever observed a romance at your current workplace?” (1 = Yes, 0 = No). The respondents were also asked to report the number of such incidents. We argue that these workplace romance measures, particularly the third-party reports seem to confine the scope of workplace romance to a particular type of sexual activity such as kissing and hugging, as the observer may not be able to inform us about the passion and the level of commitment involved in the relationship. However, we follow Pierce et al. (1996) to adopt a broader concept of workplace romance that captures workplace romance experiences of the participants, the level of commitment and the likely future of the romantic relationship. The general literature on romance relationship literature proposes that committed romantic relationships positively affect the participants’ behavior (Diamond et al., 1999; Diamond and Dube, 2002; Debrot et al., 2013; Koole et al., 2014). For example, Debrot et al. (2013) show that romantic relationship positively affects the romance participants’ behavior and also life satisfaction. Moreover, the happy productive worker hypothesis suggests that content and happy workers show improved performance in organizational settings (Iaffaldano and Muchinsky, 1985; Wright and Staw, 1999; Judge et al., 2001; Wright et al., 2007). Thus, we argue that it is likely that workplace romance positively affects employee performance.

Finally, the effects of workplace romance depend on the motive for engaging in the relationship. These may include committed love, ego satisfaction, financial rewards, adventure and sexual satisfaction (Dillard, 1987; Pierce et al., 1996). Pierce et al. (1996) suggest that the motive for engaging in a committed romantic relationship has a positive effect on employee performance. As workplace romance refers to a committed love relationship, we propose a positive relationship between workplace romance and performance.

In essence, building on the constructive effects discourse of the workplace romance (Pierce et al., 1996; Biggs et al., 2012), the impression management hypothesis (Dillard et al., 1994; Pierce and Aguinis, 2003) and perceiving workplace romance as a committed and mutually-welcomed relationship (Diamond et al., 1999; Diamond and Dube, 2002), we argue that workplace romance can have constructive effects on employee performance. Thus, we predict that workplace romance positively affects employee performance.

Hypothesis 1: Workplace romance will positively affect employee performance.

### Psychological Wellbeing as a Mediator

The concept of employee wellbeing is complex (Wright et al., 2007; de Cuper and de Witte, 2008). Scholars have proposed varying conceptualizations and models of wellbeing. A detailed analysis of these variations is beyond the scope of this paper and is not the purpose here. The literature usually focusses on three general characteristics of psychological wellbeing. First, psychological wellbeing is portrayed as a subjective experience (Diener, 1994; Wright et al., 2007; de Jong, 2014). Second, the psychological wellbeing studies focus on the affective aspects of wellbeing that include relative absence of negative emotional experiences and states, as well as the presence of positive emotional experiences and states, usually on a single axis (Wright and Staw, 1999; Cropanzano et al., 2003).

Scholarship on wellbeing also suggests that wellbeing is more than the absence of illness and negative emotions. Rather, wellbeing is conceived as positive emotions, a positive mental state and personal growth (Hofmann and Tetrick, 2003; Arnold et al., 2007). Third, literature categorizes psychological wellbeing as a context-specific wellbeing construct such as job satisfaction, and wellbeing as a global – context free – construct such as general psychosomatic complaints and life satisfaction (Warr, 1999; Grebner et al., 2005; de Jong, 2014). We follow the Diener et al. (2010) conceptualization of wellbeing that portrays a broader scope of wellbeing to encompass important aspects such as optimism, purpose and relationship quality. Moreover, this concept of wellbeing can be both contextualized and operationalized as a global construct.

Additionally, general romance literature suggests that romantic relations promote psychological wellbeing. For instance, research has shown that affectionate touch from the romance partner relieves stress and instills optimism (Ditzen et al., 2007). Affectionate touch from the romance partner manifests in the form of relaxation and good feeling, suppression of negative feelings and improved relationship quality (Burleson et al., 2007; Debrot et al., 2013; Koole et al., 2014). Floyd et al. (2009) showed that a kiss from the romantic partner reduces psychological health issues and perceived stress in life. The perception of a positive regard from the romance partner offers a sense of security and social inclusion and contributes to the reduction and prevention of stress (Ditzen and Heinrichs, 2014).

Workplace romance literature also suggests that workplace romance has a positive relationship with employee wellbeing. For instance, Pierce (1998) suggests that workplace romance creates positive energy and emotions through affective spill-over effects. Cole (2009) argues that workplace romance can be a positive force that can contribute to romance participants’ increased mental energy and life and job satisfaction. Similarly, Dillard and Broetzmann (1989) and Dillard et al. (1994) posit that workplace romance participants demonstrate enthusiasm toward work. The positive feelings originating from gratifying experiences of intact workplace romance can spill over in the form of affective effects and a positive emotional state on the romance participants’ jobs. This leads to positive attitudes and energy and improved productivity (Pierce, 1998). The workplace romance affective spill-over hypothesis (Pierce et al., 1996; Pierce, 1998) is built on the general spill-over theory (Piotrkowski, 1979; Staines, 1980) that, in its simplest form, posits that family and work experiences are correlated. That is, employees satisfied from work bring positive feelings to family (Piotrkowski, 1979). Similarly, individual life’s affective emotions and reactions can spill over to workplace (Williams and Alliger, 1994). Following this line of argument, Pierce (1998) suggests that gratifying experiences of workplace romance can have constructive effects on psychological wellbeing of the workplace romance participants. Conversely, the spill-over hypothesis also suggests that negative feelings, emotions and experiences from work and family are also correlated (Repetti, 1989). This suggests that negative experiences of workplace romance can have destructive effects on the psychological wellbeing of the workplace romance participants. However, negative repercussions of workplace romance are usually linked with dissolved, egoistic and utilitarian romances instead of intact committed romances (Dillard et al., 1994; Cole, 2009). Therefore, we propose that a gratifying intact and committed workplace romance can positively influence workplace romance participants’ psychological wellbeing.

Indeed, a number of studies suggest that workplace romance can reduce stress, anxiety and tension (Anderson and Hunsaker, 1985; Mainiero, 1986; Pierce et al., 1996; Hochschild, 1997; Cole, 2009; Biggs et al., 2012). Moreover, the general literature on romance also suggests that romantic relationships reduce depression and loneliness and improve self-esteem, optimism and a sense of competence (Keefe and Berndt, 1996; Carver et al., 2003; Simon and Barrett, 2010; Woodhouse et al., 2012). Thus, we predict a positive relationship between workplace romance and psychological wellbeing (Wright et al., 2007).

Moreover, clinical psychologists acknowledge the imperative role of the pleasantness (depression or happiness) facet of wellbeing for determining different individual outcomes. Wright and Bonett (1997) suggest that depressed individuals tend to be pessimistic and exhibit reduced motivation and self-esteem. Organizational theorists also acknowledge the effect of psychological wellbeing on work-related behaviors and outcomes and the extensive costs related to dysfunctional, psychological wellbeing (Wright et al., 1993; Quick et al., 1997; Wright and Bonett, 1997; Grawitch et al., 2007; Moradi et al., 2014). Wright and Bonett (1997) found a significant positive relationship between psychological wellbeing and employee performance. This informs the following hypotheses.

Hypothesis 2: Psychological wellbeing mediates the positive relationship between workplace romance and employee performance.

### Moderated Mediation

Literature on workplace romance highlights both constructive and destructive effects of workplace romance on employee performance. The variations in the conceptualizations and findings of the relationship between workplace romance and employee performance can be linked to a number of different factors including gender and the type of workplace romance.

Women are more cautious than men about their involvement in workplace romance (Wilson, 2015). Men tend to demonstrate a more favorable attitude toward workplace romances, whereas women have been reported to have less motivation for engaging in workplace romances (Quinn, 1977; Powell and Foley, 1998). Workplace romance experiences of men and women are different (Quinn, 1977; Powell and Foley, 1998). There are several reasons that explain gender based differences in cautiousness about, and attitudes toward, the participation in workplace romances. The status and power in society and organizations are important predictors of these differences in experiences, cautiousness, and attitudes (Wilson, 2015). Moreover, women’s involvement in workplace romance is perceived more utilitarian than men (Wilson, 2015). For example, women’ participation in workplace romance is more likely to be perceived to be linked with the achievement of carrier related objectives (Anderson and Fisher, 1991). Riach and Wilson (2007) suggest that different negative stereotypes are usually linked with women, but this is usually not the case for men. Also, women’s participation in workplace romances draws more criticism, bullying and other negative reactions from colleagues and other circles of society (Dillard et al., 1994; Wilson, 2015). This argument is particularly relevant in Pakistan, a Muslim country with a male-dominant culture, in which women’s roles and freedom are marginalized in the name of religion and culture (Shah and Shah, 2012). Therefore, women may demonstrate more cautious approach toward workplace romance. Thus, we speculate the effect of workplace romance on employee psychological wellbeing and performance may vary for men and women, where the effects for male can be stronger (Gutek, 1985; Pierce et al., 1996; Wilson, 2015).

The variations in the conceptualization of the effects of workplace romance on performance are also attributed to the romance type; i.e., lateral or hierarchical romances. Lateral workplace romance refers to a romantic relationship between peers – two employees working at the same level of an organization (Pierce et al., 1996). Hierarchical workplace romance refers to a boss-subordinate romantic relationship (Pierce et al., 1996). Pierce et al. (1996) suggest that hierarchical workplace romances negatively affect employee productivity and performance, whereas lateral workplace romances can positively contribute to employee productivity and performance. Hierarchical workplace romances are usually viewed more negatively by the organizational members (Anderson and Fisher, 1991), as they can be perceived as utilitarian and can be motivated by career advancement, ego satisfaction, and sexual experience excitement (Quinn, 1977; Pierce et al., 1996). Hierarchical workplace romances raise the issues of organizational justice, equity, dependency, and power (Mainiero, 1986; Greenberg, 1987; Foley and Powell, 1999). Destructive outcomes of workplace romances including incivility, sexually harassing behaviors and a deterioration in commitment and performance are usually linked with hierarchical workplace romances (Cole, 2009; Pierce and Aguinis, 2009). Such issues and destructive outcomes are usually not linked with lateral workplace romances (Powell, 2001; Wilson, 2015). Hierarchical workplace romances are also argued to have a negative influence on psychological wellbeing (Powell and Foley, 1998). In contrast, literature indicates that lateral workplace romance positively contributes to employee psychological wellbeing and performance (Quinn, 1977; Dillard et al., 1994; Biggs et al., 2012). Thus, we expect that the effects of hierarchical and lateral workplace romances on psychological wellbeing and employee job performance will be different, where the effect of lateral workplace romance will be stronger. The discussion in this section informs the following hypotheses.

Hypothesis 3: Gender moderates the indirect effect of workplace romance on employee performance, where the indirect effect for males is stronger.
Hypothesis 4: Workplace romance type (lateral/hierarchical) moderates the indirect effect of workplace romance on employee performance, where the effect for lateral romances is stronger.

## Research Method

### Data Collection and Analysis

We collected survey data from 311 doctors (out of total 400 distributed questionnaires) from five government teaching hospitals operating in the Punjab province of Pakistan. To reduce the common method bias, we collected data in two rounds with a lag time of 6 months between each round of data collection. In the first round, data about our independent variable, workplace romance (self-reported) was collected. Data about gender, age, experience, and the type of workplace romance (lateral or hierarchical) was also collected in the first round. In the second round, data about psychological wellbeing was collected, while data about the outcome variable, clinical performance of the doctors was collected from the respondents’ supervisors. Supervisors’ performance ratings are said to be more reliable than self-ratings (Klimoski and London, 1974; Holzbach, 1978). Data was collected between October 2014 and November 2016.

The lag time of 6 months reduces the common method bias, as employees do not usually remember their previous responses and cannot relate them to the current responses (Souitaris and Maestro, 2010). Additionally, we constrained all the variables into one factor that explained 33% of the total variance and this is well below the cut-off point of 50% (Hu and Bentler, 1999). As we will explain in the ‘measures and variables’ section, we also used the already developed and validated measurement scales for all the variables of our study.

Before starting the data collection, consent forms were sent to 800 doctors (400 male and 400 female). In the consent forms, the doctors were informed about the aims and nature of the study. The key constructs of the study such as workplace romance, hierarchical workplace romance and lateral workplace romance were also defined in the consent form. For example, we defined lateral workplace romance as a romantic relationship between two employees working at the same level of an organization, and hierarchical workplace romance as a boss-subordinate romantic relationship. They were also assured that their identity would not be revealed and that the data would only be used for academic purposes. The consent form also included the criteria for participation in the study. The criteria included ‘yes’ responses from the potential participants for the following two questions: (1) Are you currently involved in a romantic (mutually-welcomed) relationship with a colleague in your workplace (Yes or No)? (2) Are you willing to participate in two data collection rounds, separated by a time lag of almost 6 months (Yes or No)?

The conceptualization and measurement scale of workplace romance used in this study required data from respondents who could reflect on their workplace romance experiences, the level of commitment they enjoyed with their romance partners and how they envisaged their relationships would develop in the future. Therefore, we aimed to collect data from the doctors who were involved in workplace romances. After several reminders, requests and the assurance of anonymity, we were able to get a list of 406 doctors who showed consent to participate in our data collection. We could not contact six out of the 406 doctors, as they were either on leave or transferred to other locations. Therefore, in the first round, the questionnaires were distributed to 400 doctors.

Three hundred and forty-three responses were received in the first round. Of the 343 respondents who responded in the first round, we were able to contact 325 in the second round. Of these 325 received responses, 14 responses had missing data. Thus, 311 responses (133 male doctors and 178 female doctors; net response rate of over 77%) were included in the data analysis.

Supervisors’ ratings were received from the respective line managers (the managers to whom the respondents of our study were directly reporting) of our respondents (311 doctors, who had responded in both the rounds of data collection and completed the survey questionnaires in all respects). The structure of the hospitals meant that doctors reporting to a single line manager ranged from one to six. In our study, for any given line manager, the number of respondents ranged from a minimum of one to a maximum of four. Specifically, a total of 102 line managers rated the performance of 311 respondents of this study. In order to match employees’ responses from the two rounds of data collection and match employees’ and supervisors’ responses, we placed a code on each questionnaire.

The respondents’ average age was 41 years with an average of 11 years’ professional experience. Before starting the survey, the questionnaire was pilot tested with five academicians and 15 respondents. We used SEM, multi-group analysis and bootstrapping (AMOS 24.0) to test our hypotheses.

### Measures and Variables

Workplace romance as a consensual and committed relationship was measured using a modified seven-item scale (α = 0.91) from Rusbult et al. (1998). The scale contains items such as “I want our relationship to last for a very long time” and “I am committed to maintaining my relationship with my partner.” The items were measured on a five-point scale from 1 (strongly disagree) to 5 (strongly agree).

To measure psychological wellbeing, an eight-item scale (α = 0.87) was adopted and modified (to contextualize the scale in the workplace) from Diener et al. (2010). The scale included items such as “I am engaged and interested in my daily activities” and “I lead a purposeful and meaningful life.” The items were measured on a five-point scale from 1 (strongly disagree) to 5 (strongly agree).

We measured doctors’ performance using a four-item clinical performance measurement scale (α = 0.76) adopted from Carr et al. (2013). The scale included items such as ‘clinical assessment and patient management,’ ‘procedural skills,’ and ‘emergency management.’ The doctor’s performance was rated from 1 (performance below the minimum acceptable level) to 5 (performance consistently far exceeded expectations). The scale items of all the variables are presented in **Table 1**.

**Table 1:** Measurement model.

Item	Overall loadings	Male loadings	Female Loadings	Mean/SD
WR1: I want our relationship to last for a very long time.	0.49^∗^	0.44	0.55	3.49/1.31
WR2: I am committed to maintaining my relationship with my partner.	0.86	0.83	0.78	3.34/1.24
WR3: I would not feel very upset if our relationship were to end in the near future (reverse coded).	0.85	0.88	0.72	3.45/1.21
WR4: It is likely that I will date someone other than my partner within the next year (reverse coded).	0.86	0.79	0.81	3.56/1.27
WR5: I feel much attached to our relationship-very strongly linked to my partner.	0.63	0.52	0.53	3.44/1.15
WR6: I want our relationship to last forever.	0.42^∗^	0.49	0.38	3.32/1.19
WR7: I am oriented toward the long-term future of my relationship.	0.56^∗^	0.45	0.59	3.23/1.09
PWB1: I lead a purposeful and meaningful job.	0.40^∗^	0.46	0.36	3.34/1.27
PWB2: My relationships with colleagues are supportive and rewarding.	0.79	0.86	0.72	3.42/1.23
PWB3: I am engaged and interested in my daily work activities.	0.69	0.69	0.67	3.56/1.15
PWB4: I actively contribute to the happiness and wellbeing of my colleagues.	0.55^∗^	0.45	0.58	3.35/1.22
PWB5: I am competent and capable the activities that are important part of my job.	0.40^∗^	0.37	0.42	4.01/1.31
PWB6: I am a good person and live a good life.	0.72	0.69	0.75	3.98/1.21
PWB7: I am optimistic about my future career prospects.	0.93	0.91	0.93	3.51/1.12
PWB8: My colleagues respect me	0.54^∗^	0.53	0.47	3.71/1.22
JP1: How do your rate his/her clinical assessment and patient management skills?	0.66	0.62	0.69	3.44/1.22
JP2: How do you rate his/her procedural skills?	0.44^∗^	0.47	0.42	3.30/1.16
JP3: How do you rate his/her emergency management?	0.72	0.77	0.66	3.47/1.23
JP4: How do you rate his/her adverse event identification and risk minimization?	0.79	0.83	0.76	3.48/1.31

*^∗^Items were dropped; SD, standard deviation; WR, workplace romance; PWB, psychological wellbeing; JP, job performance; *n* = 311, males = 133, females = 178.*

Data about the type of workplace romance was collected by asking the respondents whether they are involved in a lateral workplace romance (1) or in a hierarchical workplace romance (2). Data about homosexual and heterosexual romances was collected by asking the respondents whether they are involved in a homosexual workplace romance (1) or a heterosexual workplace romance (2).

### Control Variables

Homosexual and heterosexual workplace romances have been reported to have different effects on employees’ work-related behaviors and performance outcomes (Cole, 2009; Wilson, 2015). Thus, we initially intended to control for homosexual/heterosexual romances. However, our sample reported only 11 homosexual romances. The sample shows little variance and restricts the potential effect of heterosexual or homosexual romances on employee performance. Thus, we did not control for homosexual/heterosexual romances.

Age and work experience may confound the results (Powell, 2001). However, age and work experience were highly correlated (*r* = 0.89, *p* < 0.05) in our sample. Thus, we controlled for age, which we perceived to have more conceptual relevance to the relationship between workplace romance and performance.

## Results

### Measurement Model

We used confirmatory factor analysis (CFA) to test the fitness of our overall (to include both males and females) measurement model with the data. Our model consisted of three latent constructs – workplace romance (WR), psychological wellbeing (PWB), and employee performance (JP) – and 19 observed variables. The items (WR1, ER6, PWB1, PWB5, and JP2) that showed either suboptimal loading were dropped from the analysis. As the purpose of this study was also to test the moderating role of gender on the indirect effect of workplace romance on employee performance, we also performed CFA separately for both males and females. The observed variables, WR7, PWB4, and PWB8 demonstrated suboptimal loadings on either males or females, and were dropped.

The fit indices of the measurement model, χ^2^ = 110.513, df = 41, χ^2^/df = 2.695 < 3, GFI = 0.979 > 0.90, IFI = 0.986, CFI = 0.986 and TLI = 0.981 > 0.95, and RMSEA = 0.043 < 0.06 show that the model has a good fit with the data.

The items, their mean values, standard deviation (SD) and standardized outer loadings are presented in **Table 1**. The values of α > 0.70 for all the variables (**Table 2**) showed a satisfactory level of internal consistency. The values of composite reliability (CR), maximum shared variance (MSV), average variance extracted (AVE), and average shared variance (ASV) of WR, PWB, and JP for overall model and males and females are presented in **Table 2**. The values of CR > 0.70 demonstrated a satisfactory level of reliability for overall model and male and female groups. Moreover, AVE > 0.50 and CR > AVE (**Table 2**) showed a satisfactory level of convergent validity.

**Table 2:** Discriminant validity.

Construct	CR	AVE	ASV	MSV	WR	PWB	JP
**Overall**							
WR	0.88	0.65	0.13	0.23	**0.81**		
PWB	0.87	0.62	0.15	0.23	0.48	**0.79**	
JP	0.77	0.53	0.05	0.08	0.18	0.28	**0.73**
**Males**							
WR	0.85	0.59	0.18	0.34	**0.77**		
PWB	0.87	0.63	0.22	0.34	0.58	**0.79**	
JP	0.79	0.56	0.06	0.10	0.12	0.31	**0.75**
**Females**							
WR	0.81	0.52	0.06	0.09	**0.72**		
PWB	0.85	0.60	0.07	0.09	0.30	**0.77**	
JP	0.75	0.50	0.03	0.04	0.15	0.21	**0.71**

*WR, workplace romance; PWB, psychological wellbeing; JP, job performance; CR, composite reliability; AVE, average variance extracted; MSV, maximum shared variance; ASV, average shared variance; *n* = 311, males = 133, females = 178. Bolded values represent the square root value of AVE.*

The square root values of AVE of WR, JP, and PWB (**Table 2**) are greater than their inter-construct correlations. Additionally, ASV, MSV < AVE. Thus, the scales we used to measure WR, JP, and PWB demonstrated an acceptable level of discriminant validity. The square root values of AVE are presented on the diagonal in the last three columns of **Table 2**, while all other values in the last three columns of **Table 2** are the values of inter-construct relations.

### Structural Model

To evaluate the structural model, first, the direct effect of workplace romance on employee performance was tested. The direct effect of workplace romance on employee performance was significant (β = 0.183, *p* = 0.000 < 0.001). The fit indices of this initial model (1), χ^2^ = 32.149, df = 13, χ^2^/df = 2.473, GFI = 0.981, IFI = 0.993, CFI = 0.993, TLI = 0.991 and RMSEA = 0.040 showed that the model has a good fit with the data. Thus, our hypothesis (1) is supported.

Then we included psychological wellbeing as the mediator in model (2). The fit indices used for the evaluation of the model (2), χ^2^ = 141.281, df = 51, χ^2^/df = 2.77, GFI = 0.976, IFI = 0.982, CFI = 0.982, TLI = 0.976, and RMSEA = 0.044 show that the role of the mediator, psychological wellbeing, is important in the relationship between workplace romance and employee performance.

**FIGURE 1:**
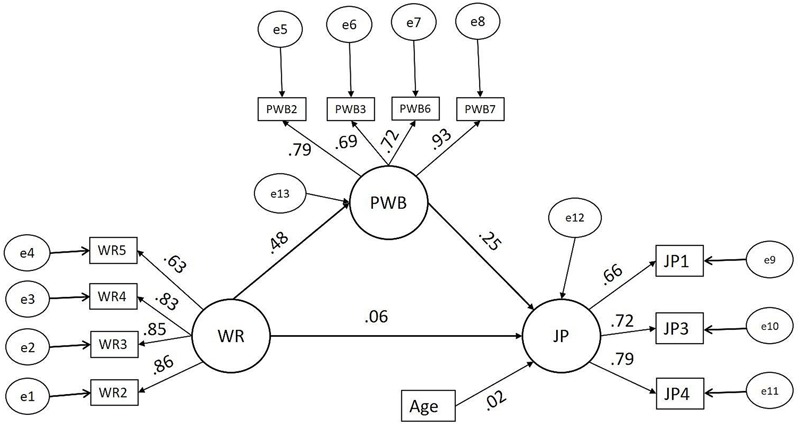
Structural model (2) – mediation model. WR, workplace romance; PWB, psychological wellbeing; JP, job performance.

The significance of psychological wellbeing as a mediator in the relationship between workplace romance and employee performance was tested using AMOS bootstrapping by specifying a sample of 2,000 at a 95% confidence interval. The model (2) is presented in **Figure 1**. The results obtained using bootstrapping are shown in **Table 3**.

**Table 3:** Direct and indirect effects and 95% confidence intervals.

Parameter			Estimate	Lower	Upper
**Model (2)**					
*Standardized direct effects*					
PWB	<—	WR	0.484^∗^	0.409	0.554
JP	<—	PWB	0.250^∗^	0.156	0.340
JP	<—	WR	0.062	-0.033	0.145
*Standardized indirect effects*					
JP	<— PWB	<— WR	0.121^∗^	0.076	0.174
**Model (A): males**					
*Standardized direct effects*					
PWB	<—	WR	0.576^∗^	0.480	0.667
JP	<—	PWB	0.367^∗^	0.201	0.516
JP	<—	WR	-0.092	-0.239	0.055
*Standardized indirect effects*					
JP	<— PWB	<— WR	0.212^∗^	0.117	0.322
**Model (B): females**					
*Standardized direct effects*					
PWB	<—	WR	0.297^∗^	0.171	0.410
JP	<—	PWB	0.180^∗^	0.078	0.287
JP	<—	WR	0.096	-0.012	0.214
*Standardized indirect effects*					
JP	<— PWB	<— WR	0.053^∗^	0.021	0.101

*^∗^Results are significant *p* < 0.05, as the confidence internal does not overall with zero. WR, workplace romance; PWB, psychological wellbeing; JP, job performance, *n* = 311, males = 133, females = 178.*

**Table 3** shows that the indirect effect of workplace romance on employee performance is significant. Moreover, the direct effect of workplace romance on employee performance is non-significant. The effect of the control variable, age (β = 0.024, *p* = 0.51 > 0.05), was non-significant. Thus, our hypothesis (2) is supported. That is, our results show that psychological wellbeing significantly mediates the positive relationship between workplace romance and employee performance.

### Moderated Mediation

To test the moderation effect of gender on the mediation model presented in **Figure 1**, we used multi-group analysis, bootstrapping (specifying a sample of 2,000 at a 95% confidence interval) techniques and χ^2^ difference test. Multi-group moderation involves the testing of structural model estimates (Hair et al., 2010). For this purpose, the moderating variable was first categorized into two groups – male (1) and female (2). Second, the unconstrained model (the model in which none of the paths is constrained for the equality of the structural weights) was estimated. Third, the constrained model was estimated by constraining the structural weights along the paths of interest to be equal between the groups (Hair et al., 2010). Finally, the χ^2^ values and degrees of freedom of the unconstrained and constrained models were compared to determine if the models are significantly different (Hair et al., 2010). The multi-group analysis function in AMOS estimates both the unconstrained and constrained models in a single step, provides the χ^2^ values and degrees of freedom of the unconstrained and constrained models and compares these values for the significance of difference between the models. The model comparison outputs showed that the χ^2^ difference between the unconstrained (χ^2^ = 209.506, df = 102) and constrained (χ^2^ = 232.753, df = 105) models was significant (χ^2^ difference = 23.247, df difference = 3, *p* = < 0.001). This suggests that gender moderates the indirect effect of workplace romance on employee performance.

**FIGURE 2:**
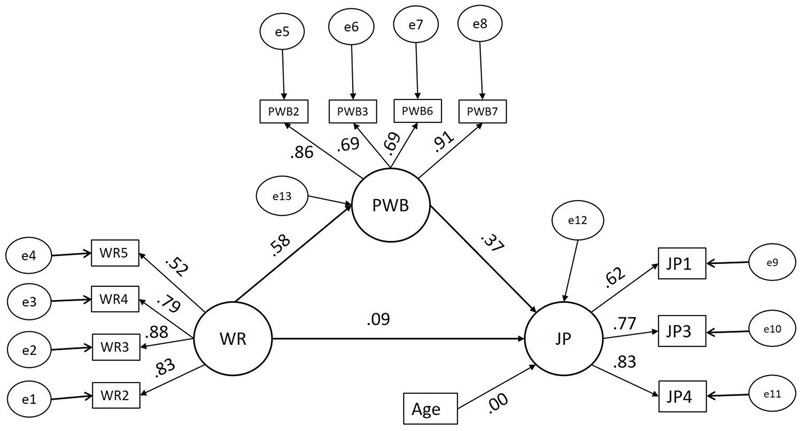
Structural model (A) – Moderated mediation model – males. WR, workplace romance; PWB, psychological wellbeing; JP, job performance, standardizes estimates shown, *n* = males = 133.

**FIGURE 3:**
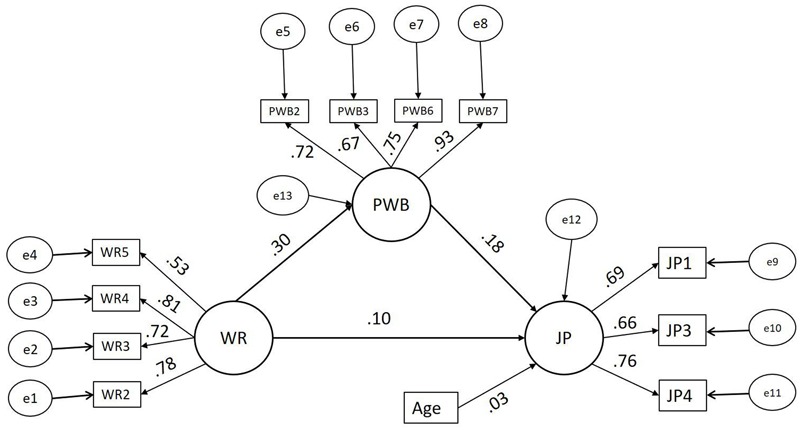
Structural model (B) – Moderated mediation model – females. WR, workplace romance; PWB, psychological wellbeing; JP, job performance, standardizes estimates shown, *n* = females = 178.

The moderated mediation model (A) for the male group is presented in **Figure 2**. The moderated mediation model (B) for the female group is presented in **Figure 3**. The bootstrapping results of model (A) and model (B) are presented in **Table 3**. The fit indices, χ^2^ = 209.506, df = 102, χ^2^/df = 2.054, GFI = 0.978, IFI = 0.986, CFI = 0.986, TLI = 0.982, and RMSEA = 0.034 showed that the model has a good fit with the data. Also, the fit indices demonstrate improvement when compared with the mediation model (2).

The bootstrapping results of models (A) and (B) presented in **Table 3** show that the indirect effect of workplace romance on employee performance is significant for both males and females. Moreover, the direct effect of workplace romance on the performance is non-significant. That is, the results show that psychological wellbeing significantly fully mediates the relationship between workplace romance and employee performance for both males and females separately. The results of the heterogeneity test showed that the indirect effect (unstandardized β = 0.179, standard error = 0.042) of WR on JP for males is significantly stronger (*z* = 2.687, *p* = 0.007 < 0.01) than the indirect effect (unstandardized β = 0.054, standard error = 0.020) of WR on JP for females. Our hypothesis (3) predicted that gender moderates the indirect effect of workplace romance on employee performance, where the effect for males is stronger. Thus, our results support hypothesis (3).

Hypothesis (4) proposed that the workplace romance type (lateral or hierarchical) moderates the indirect effect of workplace romance on employee performance, where the relationship for lateral workplace romances is stronger. To test the moderation effect the workplace romance type on the mediation model, we used multi-group analysis, bootstrapping (specifying a sample of 2,000 at a 95% confidence interval in AMOS 24.0) techniques and χ^2^ difference test. For this purpose, the moderating variable was first categorized into two groups – lateral romance (1) and hierarchical (2). Second, the unconstrained model was estimated. Third, the constrained model was estimated by constraining the structural weights along the paths of interest to be equal between the groups. Finally, the χ^2^ values and degrees of freedom of the unconstrained and constrained models were compared to determine if the models are significantly different. The χ^2^ difference between the unconstrained (χ^2^ = 215.712, df = 102) and constrained (χ^2^ = 220.98, df = 105) models was non-significant (χ^2^ difference = 5.468, df difference = 3, *p* = 0.141 > 0.001). This suggests that the workplace romance type does not moderate the indirect effect of workplace romance on employee performance.

Bootstrapping results showed that psychological wellbeing mediated the relationship between workplace romance and performance for both lateral and hierarchical romances separately. The results of the heterogeneity test showed that the indirect effect (unstandardized β = 0.128, standard error = 0.03) of WR on JP for lateral romances is not significantly different (*z* = 1.418, *p* = 0.139 > 0.05) from the indirect effect (unstandardized β = 0.072, standard error = 0.023) of hierarchical romances on employee performance. Therefore, the results do not support hypothesis (4).

## Discussion and Conclusion

### Key Findings and Theoretical Contributions

We examined the relationship between workplace romance and employee performance and also tested and confirmed the mediating role of psychological wellbeing in the relationship between workplace romance and employee performance. Based on a survey of 311 doctors from five government teaching hospitals operating in the Punjab province of Pakistan, we used SEM and bootstrapping technique to test these relationships.

Our results show that the workplace romance as a mutually-welcomed and committed relationship positively affects employee performance. This finding concurs with the literature that posits a positive relationship between workplace romance and employee performance (Dillard et al., 1994; Pierce and Aguinis, 2003).

Moreover, our study reveals that psychological wellbeing fully mediates the relationship between workplace romance and performance. In this way, our study extends the workplace romance and performance literature by showing that workplace romance positively contributes to psychological wellbeing that, in turn, positively affects employee performance. This finding concords with the literature (Dillard and Broetzmann, 1989; Pierce, 1998; Cole, 2009) that suggests a positive relationship between workplace romance and psychological wellbeing.

Our study pulls together and provides the empirical evidence of the cross-links between different important knowledge disciplines – workplace romance, psychological wellbeing, and employee performance. Our findings bring attention to the role and importance of psychological wellbeing in the social fabric of our workplace, and particularly in healthcare work, where workers’ existence, purpose and work practices are not readily analogous to those of commercial businesses (Mol, 2008). Healthcare professionals are consistently exposed to patients with varying degrees of vulnerability, unease and critical condition that remind the healthcare professionals of their own vulnerabilities and fragmentation and may incite desire for care and love. Therefore, marketized efficiency-driven policies strongly driven by surveillance, accountability and relational and emotional detachment may not work in healthcare sector (Fotaki, 2015). Also, healthcare staff’s compassion and emotions for the patient as well as for the colleagues are inevitable (Fotaki, 2015). Moreover, it is an important feature of good practice and a facilitator of reflecting and thinking rationally in the best interest of patient care (Fotaki, 2015).

Importantly, by showing that gender moderates the indirect effect of workplace romance on employee performance, we contribute to the debate on the constructive/destructive outcomes of the workplace romance. Our results show that psychological wellbeing significantly mediates the relationship between workplace romance and clinical performance for both males and females separately. However, the results reveal that the indirect effect of workplace romance on employee performance for males is significantly stronger than for females. Seen in this light, our finding agrees with Pierce et al. (1996) and Biggs et al. (2012) who posit that the effects of workplace romances may vary across gender. This finding also seems to support the stance that the cultural interpretation of Islam tends to empower men and marginalize women (Shah and Shah, 2012).

### Practical Significance

Our findings signify the importance of romance in the workplace in general, and particularly in healthcare services. Our findings suggest that workplace romances should not be perceived as inherently problematic, at least in relation to the performance of the doctors and their effects on psychological wellbeing. Therefore, policy makers and managers should not be concerned about implementing strict measures to overcome workplace romances if they deem that ethical, legal and cultural norms and performance expectations are not violated. The positive relationship between workplace romance and doctors’ performance would mean improved healthcare provision to the patients and better service to human life.

The findings suggest that employee wellbeing should be a key part of organizational policy, which is usually strongly driven by accountability and strict procedural guidelines in the healthcare sector (Rizq, 2013). Our findings laud for looking beyond the procedural strictness and obsession with regulation that seem focussed on relational detachment in the workplace and suppression of affection and love toward colleagues at work.

However, the findings of this study require profound deliberations, as workplace romances may be seen as problematic by a number of stakeholders because of their potential contradictions with the cultural, religious, ethical, and moral norms of the organization and society.

### Limitations and Future Research Directions

One of the limitations of our study is that we focussed on government hospitals in one of the provinces of Pakistan, whereas workplace romance outcomes can be different in other provinces and rural areas. The results may vary across cultures and contexts. Future studies in different cultures and contexts can enhance our understanding of the work-related outcomes of workplace romance.

Moreover, our sample involved respondents (doctors) from a profession, which is equally attractive to both males and females. Women make up almost half of the population of Pakistan. The cultural interpretation of Islam has confined women to the peripheral, domestic, and supportive roles to ascertain the male dominance in the central roles (Shah and Shah, 2012). Nevertheless, in a few professions, including the medical profession, the participation of women is encouraging. For instance, in the Punjab province, registered female doctors contribute almost 48% of the total number of registered doctors (Punjab Development Statistics, 2015). Thus, it is likely that the findings in male-dominated professions are different. The studies in male-dominated professions (de Haas and Timmerman, 2010) may offer valuable insight into the gender-based differences in the relationship between workplace romance and performance. These limitations portray an important future research agenda.

Additionally, we focused on intact workplace romances and our sample included participants who were involved in workplace romances. However, the literature suggests that a large percentage of workplace romances dissolve over time and result in negative repercussions in the form of emotional disorders, poor job performance, litigations and job loss (Pierce and Aguinis, 2009; Wilson, 2015). This suggests that, in the long run, the nature of a romantic relationship may change and dissolve in such a way that can be detrimental to the behavioral and performance outcomes of employees and organizations. This presents an important future research area that can further enhance our understanding on the nature of the effects of workplace romance.

Another limitation of our study is that we did not focus on workplace romances as extramarital affairs, which are posited as unethical and destructive for a number of stakeholders within and outside an organization (Dillard et al., 1994; Wilson, 2015). As extramarital relationships are usually against the ethical and social norms of the workplace and society, these relationships can disrupt the social climate of the workplace and society, and can attract hostility, cynicism and disapproval from peers, managers, family members, and society (Dillard et al., 1994; Pierce and Aguinis, 2009). Future research can bring to the fore the consequences of workplace romances as extramarital affairs.

Finally, we relied on self-reports for measuring workplace romance and this approach may be viewed as being problematic. Future studies may consider supervisors’ or colleagues’ reports, providing these do not violate privacy and other ethical standards.

## Ethics Statement

This study was carried out in accordance with the recommendations of the Ethical Principles of Psychologists and Code of Conduct by the American Psychological Association’s (APA). All participants gave written informed consent in accordance with the Declaration of Helsinki. The protocol was approved by the employee’s council of the participating organizations as well as the ethics committee of COMSATS Institute of Information Technology Lahore.

## Author Contributions

Definition of research objectives, models, and hypotheses: MASK, DJ, and MU. The provision of materials (i.e., questionnaires): MASK and DJ. Data collection: MU and MA. Data analysis plan: MASK, MU, and MA. Data analysis: DJ. Principal article writing: MASK and MU. Article revision and proofreading: DJ and MA. Final approval: MASK, DJ, MU, and MA.

## Conflict of Interest Statement

The authors declare that the research was conducted in the absence of any commercial or financial relationships that could be construed as a potential conflict of interest.
